# Successful Removal of a Fractured Guidewire During Percutaneous Coronary Intervention With Orbital Atherectomy

**DOI:** 10.1016/j.jaccas.2026.109241

**Published:** 2026-07-09

**Authors:** Harish Ravipati, Edo Kaluski

**Affiliations:** a Guthrie Cortland Medical Center, Cortland, New York, USA; b Guthrie Robert Packer Hospital, Sayre, Pennsylvania, USA

**Keywords:** coronary angiography, left-sided catheterization, percutaneous coronary intervention

## Abstract

**Background:**

Guidewire entrapment or fracture is a rare complication during percutaneous coronary intervention (PCI). Timely management is warranted to prevent poor cardiac outcomes.

**Case Summary:**

An 83-year-old man with coronary artery disease was referred for PCI given severe left anterior descending disease and right coronary artery disease (RCA). During PCI of in-stent restenosis of the RCA, the 0.014-inch Versaturn guidewire (Abbott) was entrapped in the coronary calcium within a prior stent, and the tip fractured. The wire entrapment led to a technically complex procedure, which was treated with orbital atherectomy and stenting of the RCA to cover the entrapped fractured wire.

**Discussion:**

Previous reports have highlighted the use of rotational atherectomy in coronary artery remodeling and the management of entrapped or fractured guidewires. This is the first reported case employing orbital atherectomy to manage an entrapped fractured guidewire within calcific in-stent restenosis.

**Take-Home Message:**

Guidewire entrapment within critical calcific lesions creates considerable difficulty, which can lead to cardiac complications, and it can be managed with orbital atherectomy and stenting.

## Graphical Abstract

**Figure:**
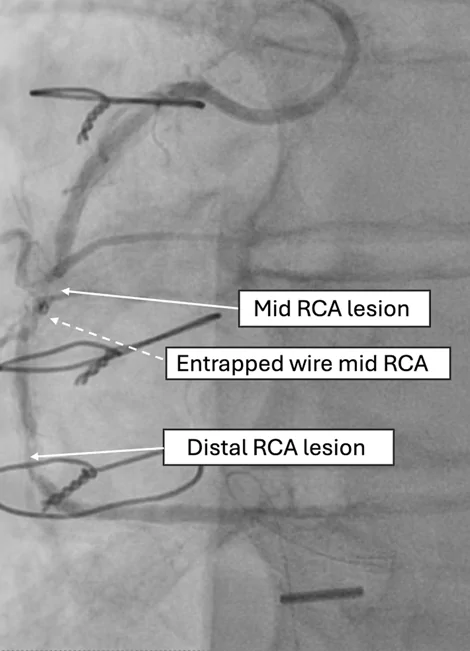

**Visual Summary:**
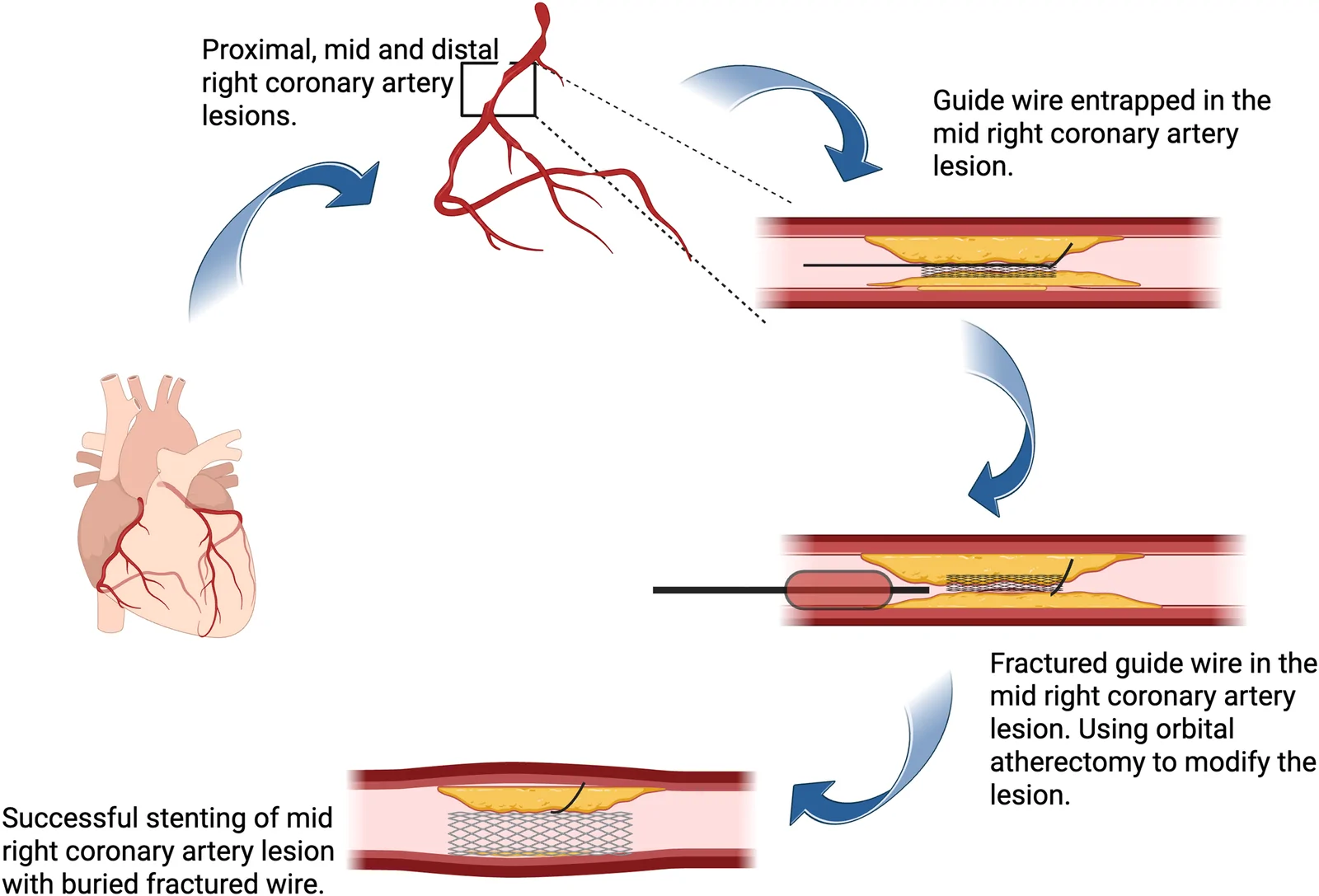
Successful Removal of a Fractured Guidewire Using Orbital Atherectomy

## History of Presentation

An 83-year-old man with a history of coronary artery disease (CAD) and coronary artery bypass grafting presented with ongoing shortness of breath. Physical examination findings included jugular venous pressure 5 to 6 cm above the right atrium, normal S1, and 2/6 late-peaking crescendo-decrescendo systolic murmur at the right upper sternal border with absent A2 and trace bilateral lower extremity edema.

**Take-Home Message**

• Guidewire entrapment within critical calcific lesions creates considerable difficulty, which can lead to cardiac complications; it can be managed with orbital atherectomy and stenting.

## Past Medical History

The patient had a history of CAD with subsequent inferior myocardial infarction and right coronary artery (RCA) stenting, coronary artery bypass grafting with left inferior mammary artery to left anterior descending artery (LAD) and saphenous venous graft to obtuse marginal and first diagonal branch, chronic heart failure with preserved ejection fraction, and severe low-flow low-gradient aortic stenosis (AS). Transthoracic echocardiogram (TTE) in April 2024 demonstrated left ventricular ejection fraction of 55% to 60%, grade 1 diastolic dysfunction, and severe AS with valve area by continuity equation 0.69 cm^2^, peak transaortic velocity was 3.6 m/s, mean and peak gradients across the aortic valve were 31 mm Hg and 50 mm Hg, respectively. Other comorbidities included obstructive sleep apnea, chronic obstructive pulmonary disease, chronic kidney disease stage IIIa, and hypertension. Hemoglobin was 17 g/dL, platelets 211,000, N-terminal pro–B-type natriuretic peptide 536 pg/mL, and creatinine 1.4 mg/dL.

## Differential Diagnosis

The differential diagnosis included worsening CAD, AS, or heart failure exacerbation.

## Investigations

Left heart catheterization in October 2024 showed patent left inferior mammary artery–to–apical LAD. The proximal LAD was 70% stenosed, the instantaneous wave free ratio was 0.89, and the first diagonal was 90% stenosed with a patent saphenous venous graft to the diagonal branch. The proximal RCA was 70% stenosed, mid-RCA was 99%, and distal RCA was 80% stenosed. There was moderate AS with a mean pressure gradient of 22 mm Hg, with normal cardiac output and cardiac index, pulmonary capillary wedge pressure, and slightly elevated right-sided pressures were noted. Repeat TTE in April 2025 showed preserved left ventricular ejection fraction at 55% to 60% with severe AS.

## Management

The patient had ongoing symptoms of shortness of breath and limited physical activity. Pulmonary function tests remained stable. TTE showed severe AS, and left heart catheterization showed moderate to severe CAD. The patient was managed medically because of ongoing symptoms and was again referred for percutaneous coronary intervention (PCI) with an intention of future transcatheter aortic valve replacement if symptoms did not resolve.

Left heart catheterization showed aortic saturation of 85%, with cardiac output of 5.6 L/min, cardiac index of 2.8 L/min/m^2^, and systemic vascular resistance of 13 Wood units. Right heart catheterization showed right atrium 0 mm Hg, right ventricle 39/6 mm Hg, pulmonary artery 39/11 mm Hg (mean: 19 mm Hg) with saturation of 66%, pulmonary vascular resistance of 1 Wood unit, and pulmonary capillary wedge pressure 12 mm Hg.

**Figure 1:**
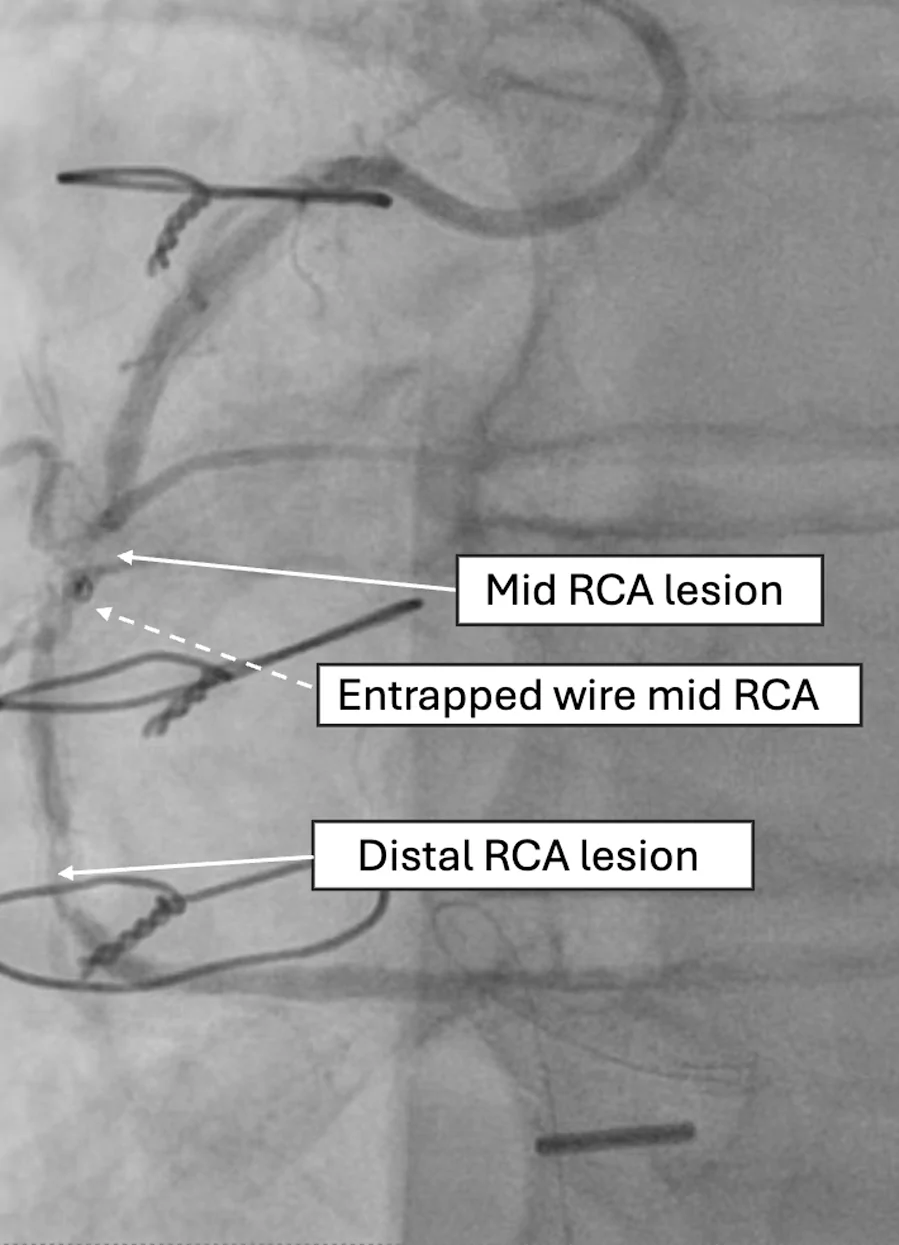
Image During Percutaneous Coronary Intervention Showing the Right Coronary Artery With Mid and Distal Lesions and an Entrapped Wire in the Mid Right Coronary Artery RCA = right coronary artery.

**Figure 2:**
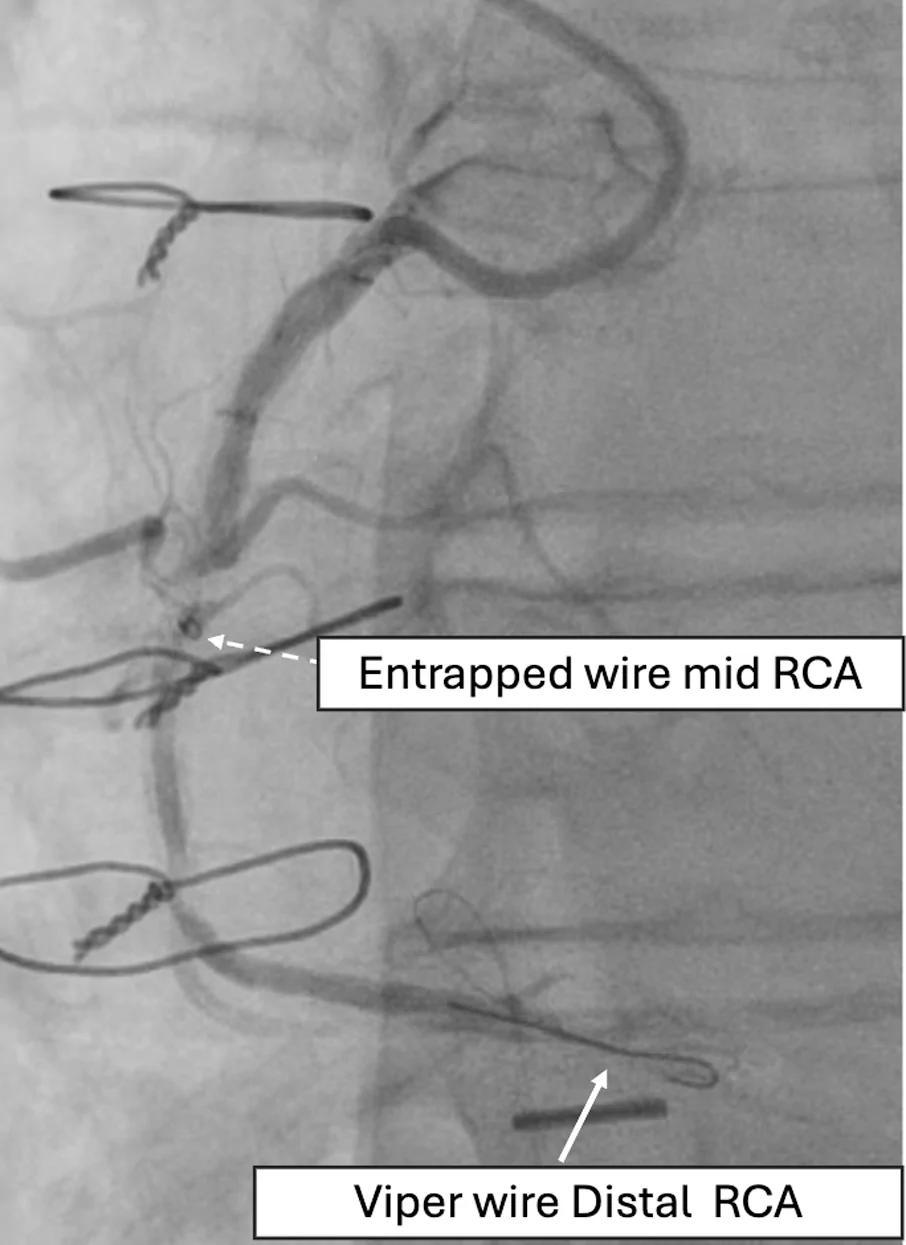
Image During Percutaneous Coronary Intervention Showing Entrapped Wire in the Mid Right Coronary Artery and ViperWire in the Distal Right Coronary Artery RCA = right coronary artery.

The RCA was dominant and engaged with an Amplatz left 0.75 guide with 6 F (Boston Scientific), and a Versaturn 0.014-inch wire (Abbott) was advanced with difficulty in the distal RCA. A 6-F Guideliner was advanced into the proximal RCA. The attempt to slightly reposition the Versaturn resulted in entrapment of the distal tip of the wire. During an attempt to withdraw the Versaturn wire, the 1-cm distal tip of the Versaturn was sheared (when compared to similar unused wire) (Figure 1). There was no uncoiling or unwrapping of the wire. RCA was successfully rewired with Fielder XT and Fielder FC (Asahi Intecc) but could not even advance any balloons of 1.25 or 1.5 mm into the lesion, which was stented and heavily calcified, despite a very supportive guiding catheter and a Guideliner. We attempted to use a snare to remove the wire remnant in the RCA, but the snare could not traverse the calcified stented lesion. The Guideliner was then pushed as far as possible to support the procedure, but even advancing it to the mid-RCA lesion did not resolve the issue. Later, the Fielder XT was switched to an orbital atherectomy (OA) ViperWire (Abbott) (Figure 2). Subsequently, we attempted OA with a 1.25-mm crown, but the orbital catheter stalled. We had to remove the OA device and wire as a single unit. We upgraded the femoral sheath from a 6-F to an 8-F sheath. The Amplatz left 0.75 guide was upgraded to 8-F. A second ViperWire and OA device were used, with the support of an 8-F Guideliner. After the RCA was rewired with the ViperWire, OA was performed at 80,000 rpm for safety of the vessel, to prevent microcavitation and vessel injury, which tend to occur more frequently at higher speeds (eg, 120,000 rpm). The OA device was removed, and a conventional wire was advanced into the distal RCA and into the posterior left branch.

**Figure 3:**
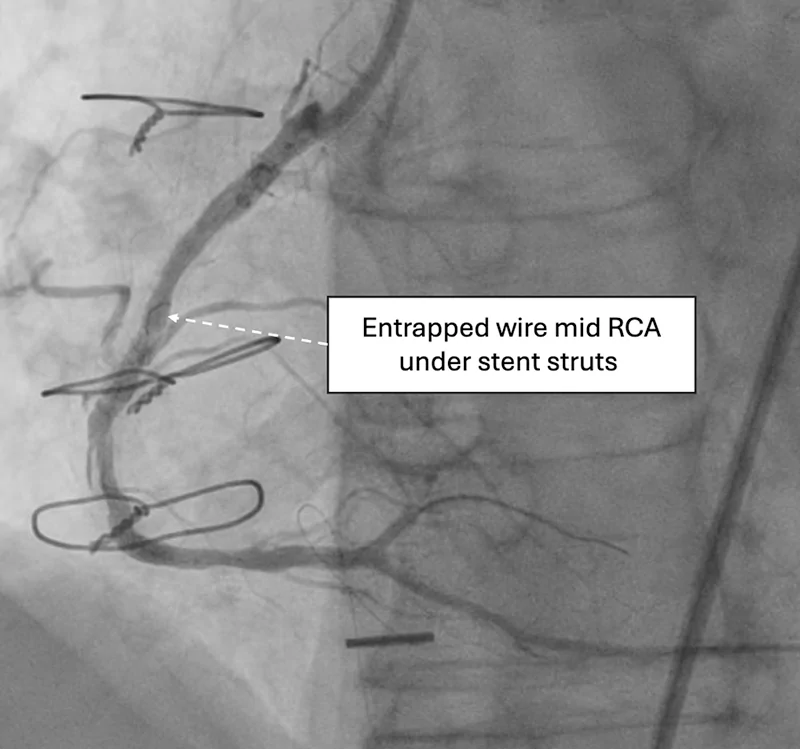
Image During Percutaneous Coronary Intervention Showing an Entrapped Wire Under Stents in the Mid Right Coronary Artery RCA = right coronary artery.

Dilatation was performed initially with an Emerge 2.75 × 30 balloon (Boston Scientific) of both the distal RCA lesion and the mid RCA lesion. Subsequently, we used an Euphora 3 × 27 balloon (Medtronic) to inflate the distal lesion in the mid-RCA lesion. Within the distal lesion, a Xience Skypoint 2.5 × 23 stent (Abbott) was deployed, and in the mid-RCA, an Onyx Frontier 3 × 38 (Medtronic) was deployed. The balloon of the Onyx Frontier was used to postdilate the proximal part of the Xience Skypoint stent (Figure 3). Finally, we achieved a normal flow in the RCA, and the patient was completely asymptomatic after the procedure (Video 1). The patient remained hemodynamically stable, the right common femoral sheath was removed, and hemostasis was achieved. Dual antiplatelet therapy was recommended. A total of 168 mL of contrast was recorded however, and some of this contrast was lost in multiple equipment changes.

## Outcome and Follow-Up

After 6 months, the patient underwent transcatheter aortic valve replacement with a 34-mm Evolut FX+ valve (Medtronic) and is currently doing well without complications.

## Discussion

Entrapment or fracture of the guidewire during PCI is rare, and this happens because of severe coronary atherosclerosis and calcification, chronic total occlusion, or prior in-stent stenosis. Excessive rotation with the tip in the calcified lesion is the most common cause of fracture of the guidewire during PCI.^1^

Entrapment or fracture of the guidewire can lead to fatal complications such as ischemia, infarction, coronary artery dissection, perforation of the vessel, and hemopericardium if not addressed during the attempt to retrieve the entrapped wire.

When a wire gets entrapped, the initial strategy should be to advance a balloon or a microcatheter to release the entrapped wire using gentle tags.

Severed wire mandates assessment of the wire removed from the body to assess (by comparing similar unused wire) the length of the missing segment and whether the wire was unraveled.

The most important factors that drive management include the patient's hemodynamic status, the involved vessel, and the length of the fractured guidewire. The most common techniques employed for retrieving a fractured guidewire are snaring, using a bioptome, and the double-wire technique (twisting the 2 wires to trap the fractured guidewire). If the wire cannot be retrieved and the missing length is known, stenting with the fractured guidewire and, in rare cases, open cardiac surgery should be considered.^1^

Sometimes it can be difficult to snare the sheared wire when embedded in coronary calcium deposits or underneath a stent. In that case, vessel preparation with balloon angioplasty should be considered. It is recommended not to use excessive force to remove the fractured segment of the guidewire to avoid perforation or dissection of the coronary artery.

Pushing the fractured sheared wire to the distal part of the coronary artery during PCI and stenting can reduce the extent and impact of thrombotic ischemic events related to the retained wire.^2^

OA, rotational atherectomy (RA), and excimer laser^3^ can be used to alleviate wire entrapment after successful rewiring.^4^

Several investigators have reported using RA to facilitate the removal of an entrapped guidewire or cutting the guidewire in severely calcified coronary lesions.^5^, ^6^, ^7^ In this patient, we used OA to penetrate calcific in-stent restenosis and did not use RA, as OA has a lower chance of burr entrapment and leads to better revascularization. To our knowledge, this is the first case report to use OA to facilitate management of a sheared guidewire. OA use within in-stent restenosis is considered “off-label.”

When the fractured guidewire is managed with stenting, intravascular ultrasound or optical coherence tomography is useful to determine the length of the entrapped or fractured guidewire and confirm that the guidewire margins are covered by the stent. Intravascular ultrasound was not available on the day of the procedure, and optical coherence tomography was not performed to limit the contrast; these are limitations to this report.

If any retained equipment remains within the patient's body, an immediate report to the patient and family should be made and the safety event should be documented by the physician and facility and reported to the state.

## Conclusions

Guidewire fracture during PCI can result in serious complications if not managed properly. While RA is the most frequently reported technique for fractured guidewire management, this case emphasizes the innovative use of OA in complex coronary procedures.

## Funding Support and Author Disclosures

The authors have reported that they have no relationships relevant to the contents of this paper to disclose.

## Appendix

Video 1 Video During Percutaneous Coronary Intervention Showing Successful Placement of a Stent Over an Entrapped Wire in the Mid Right Coronary Artery
